# Succeeding with prolonged usage of consumer-based activity trackers in clinical studies: a mixed methods approach

**DOI:** 10.1186/s12889-020-09406-w

**Published:** 2020-08-27

**Authors:** André Henriksen, Anne-Sofie Sand, Trygve Deraas, Sameline Grimsgaard, Gunnar Hartvigsen, Laila Hopstock

**Affiliations:** 1 Department of Community Medicine, UiT The Arctic University of Norway, Tromsø, Norway; 2 Department of Health and Care Sciences, UiT The Arctic University of Norway, Tromsø, Norway; 3 Department of Computer Science, UiT The Arctic University of Norway, Tromsø, Norway

**Keywords:** Actigraphy, Human activity, Activity trackers, Motor activity, Intervention study, Clinical trial, Polar M430

## Abstract

**Background:**

Lack of physical activity (PA) is a risk factor for death and non-communicable disease. Despite this, more than one fourth of adults worldwide do not follow PA guidelines. As part of a feasibility study to test a complex intervention for increasing PA, we included a consumer-based activity tracker (AT) as a tool to measure PA outcomes and to track heart rate during exercise sessions. The aim of the present study was to identify factors that increase wear time when using a consumer-based AT for monitoring of participants in clinical research.

**Methods:**

Sixteen participants aged 55–74 years, with obesity, sedentary lifestyle, and elevated cardiovascular risk were recruited to a 12-month feasibility study. Participants wore a Polar M430 AT to collect continuous PA data during a six-month intervention followed by 6 months of follow-up. We performed quantitative wear time analysis, tested the validity of the AT, and completed two rounds of qualitative interviews to investigate how individual wear-time was linked to participant responses.

**Results:**

From 1 year of tracking, mean number of valid wear days were 292 (SD = 86), i.e. 80%. The Polar M430 provides acceptable measurements for total energy expenditure. Motivations for increased wear time were that participants were asked to wear it and the ability to track PA progress. Perceived usefulness included time keeping, heart rate- and sleep tracking, becoming more conscious about day-to-day activity, and improved understanding of which activity types were more effective for energy expenditure. Sources of AT annoyance were measurement inaccuracies and limited instruction for use. Suggestions for improvement were that the AT was big, unattractive, and complicated to use.

**Conclusions:**

Adherence to wearing a consumer-based AT was high. Results indicate that it is feasible to use a consumer-based AT to measure PA over a longer period. Potential success factors for increased wear time includes adequate instruction for AT use, allowing participants to choose different AT designs, and using trackers with accurate measurements. To identify accurate trackers, AT validation studies in the target cohort may be needed.

**Trial registration:**

U.S. National Library of Medicine, Clinical Trial registry: NCT03807323; Registered 16 September 2019 – Retrospectively registered.

## Background

The World Health Organization recommends at least 150 min of moderate physical activity (PA) or 75 min of vigorous PA each week for adults [1]. Physical inactivity is a leading risk factor for death and a range of non-communicable diseases, including cardiovascular disease, diabetes, and some cancers [2]. Worldwide, in the adult population, 23% of men and 32% of women were physically inactive in 2016 [3–5]. Physical inactivity is more prevalent in high-income countries, and together with obesity, they are increasing globally [3, 4]. At the population level, increased PA provides health and economic benefits [6], and achievement of the PA recommendations has shown to reduce both cardiovascular disease mortality and total mortality [7].

Most lifestyle intervention studies use traditional research instruments (e.g. accelerometers, pedometers, doubly labelled water, and calorimetry) for objective PA and energy expenditure (EE) data collection [8], but the number of studies using consumer-based activity trackers are increasing [9]. Validation studies on such activity trackers show different results, but several recent reviews show that some metrics for some activity trackers are accurate enough to measure PA in research settings [9–13]. In addition, a recent meta-analysis by Brickwood et al. [14] indicates that including an activity tracker as part of a PA intervention may increase PA participation through self-monitoring as well as assist researchers in participant monitoring. This is also supported by earlier systematic reviews, where De Vries et al. [15] found an increase of PA in adults with overweight and obesity, and Lewis et al. [16] found similar findings among intervention studies on adults.

However, few studies utilizing activity trackers use tracker output as outcomes, and recording time is mostly limited to the intervention period [8]. Exceptions include Schrager et al. [17] who used a Fitbit Flex to collect PA over 1 month (secondary outcomes), Carmichael et al. [18] who used a Garmin Vivofit 3 to collect PA (primary outcome) for up to 1 month of follow-up, and Patel et al. [19] who used a Fitbit Flex in a 12-week intervention, with 12-weeks of follow-up. Although long time follow-ups with consumer-based activity trackers are uncommon, such studies are likely to increase in frequency going forward. For instance, Halse et al. [20] are planning an RCT where participants will be asked to wear an activity tracker for 6 months as part of an intervention, with six additional months of follow-up. Similarly, Maxwell-Smith et al. [21] planned a 12-week RCT, where participants would wear a Fitbit Alta, with 12 additional weeks of follow-up. Although Fitbit-results for the follow-up period are not yet reported, results from this intervention period have been published [22].

Measuring long-term effects of a PA intervention by requesting participants to return for additional measurements several months after intervention end, can be expensive, time consuming, and add to the participant burden. To understand the long-term effect of PA interventions better, future research should include activity trackers and collect PA data during- and beyond- the intervention period. There is a need to identify success factors that can contribute to the adaptation of this approach. Phillips et al. [23] identified a range of challenges associated with using activity trackers in research. They grouped challenges into participants’ challenges, challenges with the research setting, and challenges with the activity tracker.

In the planning of a randomized controlled trial (RCT), the RESTART trial, with a complex lifestyle intervention for lasting lifestyle changes, we conducted a feasibility study that included a Polar M430 (Polar oy, Finland) activity tracker to track PA for 1 year. The Polar M430 was chosen because it was recently released (2017), claimed high pulse sensor accuracy, and had an acceptable price.

Having access to both quantitative and qualitative data from the same study gives an opportunity to gain a more complete understanding of the research topic by comparing and combining different perspectives [24]. To look further into some of the areas identified by Phillips et al. [23], we used a qualitatively driven mixed methods approach where we analysed qualitative participant interviews together with an analysis of relevant quantitative PA recordings. In this paper, we describe our findings and provide recommendations for future research.

The aim of the present study was to identify factors that increase wear time, in terms of daily wear adherence and prolonged usage, when using a consumer-based activity tracker for participant PA monitoring in clinical research.

## Method

### Participant characteristics

#### Sample

For the feasibility study we invited 75 randomly selected participants from the seventh wave of the Norwegian population based Tromsø Study [25]. Inclusion criteria were age ≥ 55 years, body mass index ≥30 kg/m^2^, self-reported sedentary lifestyle, and increased cardiovascular risk. Sixteen participants (participation 21%) responded and were recruited for a 12-month feasibility study on lasting life-style change, comprising a six-month exercise intervention with 6 months of follow-up.

#### RESTART feasibility study

Participants in the feasibility study were exposed to a 22-week intervention of two 1-h group-sessions per week with instructor-led gradually intensified exercise sessions (endurance and strength), three 2-h group counselling sessions with nutritionist (Nordic Nutritional Recommendations [26]) and psychologist (Implementation Intention-based strategies [27]). Participants wore a Polar M430 activity tracker during the intervention period and for 6 months of follow-up. The activity tracker was used for participant monitoring and to allow participants to self-monitor heart rate during training sessions. The activity tracker was *not* used as a tool for behaviour change. The primary aim of the feasibility study was to examine whether the intervention was feasible to progress to a definitive RCT, regarding recruitment, adherence, and side effects. Participants received written and oral instructions on how to wear the activity tracker. Details about the feasibility study are described elsewhere [28].

### Polar M430

#### Physical activity recording

We equipped participants with a Polar M430 activity tracker 1 week before intervention start and instructed them to wear it for the duration of the intervention study (i.e. 6 months). Participants also wore an ActiGraph for 8 days at baseline and 8 days at the end of the six-month intervention. For each participant, we therefore recorded up to 16 days of simultaneous measurements with the ActiGraph and the Polar M430. ActiGraph output was used to monitor change in PA and to test the validity of the Polar M430 in the present cohort for relevant variables (i.e. MVPA, steps, and total energy expenditure (TEE)).

#### Instruments

The Polar M430 was released in 2017. It has a six LED (light-emitting diode) wrist-based photoplethysmography sensor, i.e. optical pulse sensor, and a 50 Hz triaxial accelerometer for tracking PA. It is waterproof, weighs 51 g, has up to 20 days of battery life, and cost 150 USD. In a previous study we have shown that the Polar M430 gives valid results for TEE in a wider age- and weight-range, when compared to a hip-worn ActiGraph wGT3X-BT accelerometer (ActiGraph, Pensacola, FL, USA) [29]. The same study shows that although correlations are strong for moderate-to-vigorous physical activity (MVPA) and steps, average error is high, and researchers should be careful to use these variables to infer PA levels.

The ActiGraph is extensively used in PA research and is considered valid for PA intensity [30], step counting [31], and EE recording [32]. The ActiGraph (firmware version 1.9.2) was setup using ActiLife version 6.13.3 (ActiGraph, Pensacola, FL, USA). Output variables were generated using ActiLife. MVPA variables were calculated using triaxial activity count cut-offs at 2690 or above, as suggested by Sasaki et al. [13]. Steps were internally calculated by the ActiGraph and exported directly (through ActiLife). Activity EE variables were calculated using “Freedson VM3 Combination ‘11” (Sasaki 2011 [13] + Williams Work-Energy), and converted to TEE by adding resting energy expenditure (using the Schofield equation [33]) and 10% of TEE to account for dietary induced thermogenesis.

#### Polar M430 setup and usage

For each participant we created a de-identified account on Polar Flow [34], Polar’s online cloud storage solution, containing only demographic data (i.e. sex, year of birth, weight, and height). No identifiable information was stored on the accounts, and participants did not have access to account credentials. Since we did not want activity tracker feedback to affect participant behaviour, all notifications and feedback messages were disabled, except sleep, which was impossible to disable. The Global positioning system (GPS) was disabled to reduce battery consumption and for privacy reasons. We initially asked participants to wear the activity tracker for the duration of the study (i.e. 6 months) and to wear the tracker all day and night (24 h/day). They were told to take the activity tracker off during sleep if they experienced any discomfort.

Due to the long recording period, we asked participants who owned a smartphone to install the Polar Flow mobile application on their private smartphones. Polar Flow is used to transfer data between the activity tracker, a smartphone, and Polar’s online cloud storage. We assisted participants with connecting the activity tracker to their smartphone and aided in any issues related to the activity tracker throughout the study period.

We instructed participants to initiate data synchronization (between activity tracker and smartphone) and charging every Sunday. This bring-your-own device (i.e. smartphone) approach has shown to improve the experience and engagement of participants [35]. For participants who did not own a smartphone, we linked their activity tracker to a project smartphone. Since only five activity trackers had to be connected to the project smartphone, we did not encourage pairing with other private devices (e.g. laptop). Data synchronization between the project smartphone and activity trackers were initialized every few weeks during the weekly exercise sessions.

The first author met with participants regularly to assist in connectivity issues with the activity tracker. During these sessions, spontaneous discussions between the researcher and participants about the activity tracker occurred. Relevant information from these discussions is reported and addressed in the discussion together with other experiences from the researcher perspective.

After intervention end, we asked participants to continue to wear the Polar M430 for an additional 6 months, for a total wear time of 12 months. Participants without a smartphone meet with a researcher every 2–3 months in the follow-up period to download data from the activity tracker. After study end, we offered the Polar M430 to the participants for their private use. Participants were only informed after study end that they would receive the activity tracker. We collected no further data after the handover.

### Participant perspective

To gain a deeper understanding of participant experiences with the Polar M430, we used a qualitative approach as qualitative methods are well suited for accessing participants experiences and perceptions [36]. We performed semi-structured interviews, as described by Kvale and Brinkmann [37]. All participants took part in individual interviews at two time-points, mid-way in the intervention and 6 months after intervention end. Interview guides were developed and used during the interviews to secure that all relevant aspects were covered. An excerpt of the interview guides, with questions related to activity tracker experiences, is given in in Table 1.

**Table 1:** Excerpt from interview guides with questions related to the activity tracker

Interview	Number	Question
Mid-way	1	How was your experience with using the activity tracker?
6 months after	2	How did you use the activity tracker? (Only during workouts, or also other times? Pulse zones? As a watch?)
6 months after	3	Did the activity tracker motivate you to work out more often? Harder?
6 months after	4	Was there anything special about the activity tracker that made you more motivated?
6 months after	5	What motivated you to wear the activity tracker (for an extended period)?
6 months after	6	Is there anything you wish was possible with the activity tracker, which could have motivated you to wear it longer?

### Analysis

Participant characteristics were described descriptively. In addition, we included a comparison of responders and non-responders, using data registered at the seventh wave of the Tromsø Study. We downloaded daily values for steps, TEE, MVPA, and hours of wear time from the Polar M430, and analysed hours of wear time to define valid days for the full year of recording. A day was considered valid if the activity tracker had at least 10 h of wear time [38]. Wear time was analysed descriptively, reporting valid days (percentage of 1 year) for each participant, mean number of valid days, and number of valid days for participants who used the activity tracker for the whole 12 months of recording. In addition, wear time was analysed with participant comments.

As suggested by Phillips et al. [23], we also tested the validity of the Polar M430 to check whether it was valid in the current cohort of participants. We used repeated measures correlations [39], with bootstrapping, to calculate correlations between the Polar M430 activity tracker and the ActiGraph wGT3X-BT accelerometer. We used correlation cut-offs suggested by Evans [40], i.e. very weak: < 0.2, weak: 0.2–0.4, moderate: 0.4–0.6, strong: 0.6–0.8, and very strong: > 0.8. We also calculated mean absolute percentage error (MAPE) for each variable, using 10% error as cut-off for acceptable error in free-living studies. Finally, we used Bland-Altman limits of agreements to assess consistency between instrument outcomes [41]. Statistical analyses were performed using R version 3.5.3.

The second author performed the verbatim transcriptions of the *mid-way interview* audiotaped sessions, while a professional firm (Digforsk AS) performed the transcriptions of the *six-months after* audiotaped sessions. We used the computer software QSR NVivo 12 Plus (QSR International, Pty Ltd) as a tool for structuring data in the analysis process. We used thematic analysis when identifying and reporting themes and patterns in the data, a widely used method among health researchers [36]. We used an inductive and semantic approach to identify themes, to allow the themes to emerge from the data and to identify participant’s opinions. To identify patterns in the text, we used the six steps defined by Braun and Clarke [42] for thematic analysis: data familiarization, initial coding, generating themes, reviewing themes, defining and naming themes, and writing up report. Comments mentioned by only one participant were given equal weight as comments mentioned by multiple participants [43]. Coding was done by the first and second author and later harmonized through discussion. Analysis was done by the first author, and thoroughly reviewed by the second and last author. Quotes used in the manuscript were translated from Norwegian.

Coding was done in three iterations. The first iteration was done on paper and resulted in many partly overlapping codes. The second iteration was done in NVivo, where we merged initial codes into the following 11 themes: 1) metric inaccuracy, 2) elements that triggered irritation, 3) tracker visual design (look and feel), 4) tracker practical design (ease of use), 5) motivation for usage, 6) effect of using the tracker, 7) how tracker was used, 8) why the tracker was used, and comments on available metrics, including 9) sleep, 10) pulse, and 11) PA. These were further refined into the four final themes: motivation, activity tracker usefulness, activity tracker annoyances, and activity tracker improvements. Quotes are tagged with sex, age group, and whether they owned a smartphone or not.

## Results

### Participant characteristics

Among the 16 participants, 11 (70%) owned a smartphone and could connect their phone to the activity tracker. Participant characteristics at baseline are given in Table 2. Activity tracker recording was performed between October 2017 and September 2018. An overview of responders and non-responders, using recorded data from the seventh wave of the Tromsø Study, held approximately 2 years before the RESTART feasibility study, is given in Table 3.

**Table 2:** Participant characteristics at baseline. The RESTART feasibility study 2017–18

Characteristics	Value
Age in years, mean (SD)	66.1 (5.8)
Smartphone owner, mean age (SD)	65.2 (4.8)
Not smartphone owner, mean age (SD)	68.2 (7.8)
Male sex, % (number)	68.8 (11)
Body mass index, kg/m^2^, mean (SD)	35.6 (5.3)
Current smoking^a^, % (number)	13 (2)
High total cholesterol, % (number)	50 (8)
Low HDL (high-density lipoprotein) cholesterol, % (number)	25 (4)
Hypertension, % (number)	19 (3)

*Current smoking* Self-reported daily smoking, *High total cholesterol* Total cholesterol ≥5 mmol/L; *Low HDL cholesterol* HDL cholesterol < 1.3 (women) or < 1.0 (men) mmol/L, *Hypertension* Blood pressure ≥ 140/90 mmHg, *SD* Standard deviation. ^a^missing values: 1 participant

**Table 3:** Descriptive characteristics by attendance. The seventh wave of the Tromsø study

Characteristics	Attended the pilot	
	No	Yes
Number of participants	59	16
Age in years, mean (SD)	65.3 (5.7)	64.1 (5.8)
Male sex, % (number)	76.3 (45)	68.8 (11)
Body mass index, kg/m^2^, mean (SD)	34.0 (3.5)	36.2 (5.8)
Current smoking, % (number)	27.1 (16)	18.8 (3)
Total cholesterol, mmol/L, mean (SD)	5.8 (1.1)	5.5 (1.2)
HDL (high-density lipoprotein) cholesterol, mmol/L, mean (SD)	1.3 (0.5)	1.2 (0.3)
Systolic blood pressure, mmHg, mean (SD)	151.7 (18.3)	144.4 (15.4)

*Current smoking* Self-reported daily smoking, *SD* Standard deviation

### Polar M430

#### Wear time

**Fig. 1:**
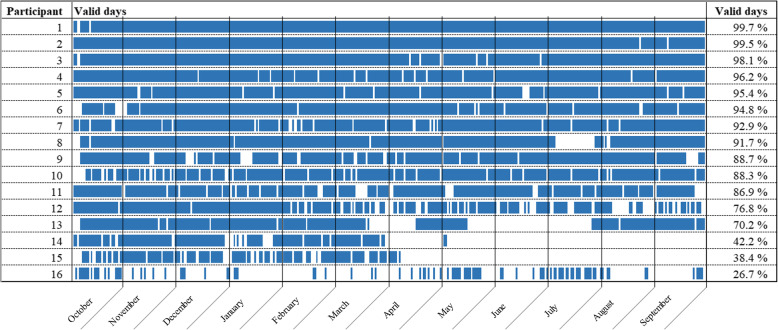
Activity tracker wear time for 1 year of recording

From the available 365 days of tracking, when including all 16 participants, mean number of valid days was 292 (SD = 86), i.e. 80%. Half of the participants had 30 or less non-valid days for the whole year of recording. Two participants (number 14 and 15 in Fig. 1) stopped using their activity tracker at the end of the intervention (after 6 months of wear time). Mean number of valid days for the whole year, when excluding these participants, was 313 (SD = 69), i.e. 88%. We observed no difference in wear time between the different months, except one participant (8) who stopped using the activity tracker during the summer holiday (July), and one participant (13) who mostly stopped using the activity tracker after the intervention but resumed wearing it after the summer months. An overview of valid days for all participants for the whole year of recording is given is Fig. 1.

The two participants who terminated use of the activity tracker after 6 months reported similar reasons for this. One participant reported being very conscious about wearing the activity tracker during the intervention and said that she became more disciplined by wearing it, which resulted in an increase in motivation. However, after the intervention ended, she “*just felt done with it*” *(Participant 10, female 70–80, smartphone).* Two specific reasons were that it was too complicated, and she had trouble with the connected smartphone, and therefore did not have easy access to all the metrics. As stated in the interviews, “*I did not see the results I wanted on my iPad* … *my daughter has a watch I like better … it is simpler*” *(Participant 10, female 70–80, smartphone).* The other participant reported mainly using the activity tracker as a tool to keep track of pulse zones during instructor led exercise sessions: “*you were told to increase your heart rate by an amount, and then you could look at the watch*” *(Participant 14, male 60–70, no smartphone)*. In addition, he did not have a connected smartphone, and felt the activity tracker was too complicated, especially without access to the instruction manual. “*When you don’t know … how to use the watch … if I had the instruction manual I could see [how to use it]” (Participant 14, male 60–70, no smartphone)*.

#### Polar M430 validity

We used output from overlapping days of Polar M430 and ActiGraph usage to test the validity of the Polar M430 in the present study. One participant did not wear both devices simultaneously and were excluded from analysis. Remaining participants had 8 to 16 valid days of simultaneous recordings. All analyses are based on data from 203 days of measurements distributed among 15 participants.

We found a strong correlation between the ActiGraph and the Polar M430 for step count, and a moderate correlation for MVPA and TEE. On average, the Polar M430 over-reports steps and time in MVPA, and under-reports TEE. Only TEE had a borderline acceptable MAPE. Details for each variable are given in Table 4.

**Table 4:** Mean data for Polar M430 and ActiGraph, and correlation, *p*-value, mean absolute percentage error, and Bland-Altman mean difference and limits of agreements (LoA), for steps, moderate-to-vigorous physical activity, and total energy expenditure. Person-days: *n* = 203

Variable	Polar	ActiGraph	Correlation (95% CI)	***P***-value	MAPE	Mean difference	Lower LoA	Upper LoA
Steps	8956 (5106)	5165 (3230)	0.625 (0.44, 0.70)	< 0.001	119.5%	3791	− 4860	12,442
MVPA	143 (97)	44 (32)	0.495 (0.31, 0.53)	< 0.001	373.9%	99.0	−80.4	278.4
TEE	2868 (581)	2967 (458)	0.446 (0.50, 0.69)	< 0.001	10.6%	− 98.7	− 948.5	751.1

Numbers are means (standard deviations). *MVPA* Moderate-to-vigorous physical activity, *TEE* Total energy expenditure, *Correlation* Repeated measurement correlation with 95% confidence interval, *MAPE* Mean absolute percentage error, *LoA* Limits of agreement

### Participant perspective

We grouped comments into four themes: Motivation, activity tracker usefulness, activity tracker annoyances, and activity tracker improvements.

#### Motivation

This theme explores if and how participants were motivated by wearing an activity tracker during- and after the intervention. Some participants mentioned the activity feedback from the activity tracker, and the possibility of directly observing progress, as the primary motivation to wear it for such a long period. For these participants, this feedback was an opportunity to push themselves harder, especially during the instructor led exercise session.

One participant stated that being able to measure progress, when she did not think there would be any progress, was very motivating and gratifying, and stated: “*I reached my goals … it was very gratifying … I did not think I would [reach my goals]*” *(Participant 12, female 70–80, no smartphone).* Another participant highlighted that the ability of using the activity tracker to push himself into working harder and harder each session was motivating, and said that “*It was interesting to follow progress, … I have never used this [technology] before, … nice to observe that … yes, now I have pushed myself*” *(Participant 2, male 50–60, smartphone).*

During ad-hoc conversations throughout the intervention period, many participants stated that they were happy with being invited to the project and wanted to contribute to the research by sharing their data. This was also confirmed in the interviews, where several participants indicated that an important reason for wearing the activity tracker for such a long period was that they were asked to do it. This willingness to share was expressed by several participants: “*We were asked to wear it ... I though it is only fair [for the benefit of the study]” (Participant 4, female 60–70, smartphone),* “*I know how important research is ... so that you will get reliable data* … *I was willing to make the ‘sacrifice’ for you and the research” (Participant 13, male 60–70, smartphone),* and *“No, I didn’t (when asked if he reviewed recorded data), I just let it [the activity tracker] do what it was supposed to do [record data] and I just did what I was supposed to do [share data]” (Participant 3, male 70–80, smartphone).*

#### Activity tracker usefulness

This theme encapsulates how and why participants used the activity tracker, as well as their perceived effects of using it. Most participants reported mainly using the activity tracker to get continuous feedback on heart rate during instructor led workouts. In addition, it was also used as a timepiece, and some used it as a tool for measuring sleep and tracking PA, during and after the intervention.

One participant highlighted the usefulness of the activity tracker by saying, “*I had to pay attention to how I performed, so I could increase resistance to get to the [heart rate] level I was supposed to be at” (Participant 1, male 60–70, smartphone).* However, not all who said they used it to track changes during a workout payed much attention to it, as illustrated by one participant who said, “*I didn’t put too much into it, but it was fun to keep track [of the activity]*” *(Participant 2, male 50–60, smartphone).*

In addition, many participants used it as a timepiece, and replaced their existing wristwatch with the Polar M430 to accommodate the study. One participant stated that he “*only used it as a watch” (Participant 13, male 60–70, smartphone)* and some simply answered “*Yes” (Participant 6, male 50–60, no smartphone. Participant 16, female 70–80, no smartphone),* when asked in a follow-up question if they simply used the watch as a timepiece.

An often-mentioned useful feature was the ability to track sleep quality and sleep interruptions during the night. For some this was an acknowledgement of what they already knew about their sleep patterns, prompting responses like “*I look at sleep … I am awake a lot*” *(Participant 8, male 60–70, smartphone)* and “*I can see how little sleep I get*” *(Participant 6, male 50–60, no smartphone)*. For others it constituted a source of confusion because the activity tracker was perceived as inaccurate, resulting in quotes like “*tracking sleep … but I don’t always think it is accurate*” *(Participant 15, female 60–70, smartphone).*

The reported effects of wearing the activity tracker were different for most participants, and only a few mentioned specific behavioural changes because of the activity tracker. However, one participant said, “*I became more disciplined*” *(Participant 10, female 70–80, smartphone).* Another participant mentioned that he became more conscious about daily activity levels and which types of activity that were effective and stated, “*I am more conscious about moving more while at work* …. *I take the stairs instead of the escalator*” *(Participant 11, male 60–70, smartphone)*, and “… *more aware of what is effective and what isn’t*” (*Participant 11, male 60–70, smartphone).* One participant highlighted this learning effect by saying, “*I learned something from the watch. Things that I thought was [effective] … the watch showed me that it actually wasn’t*” *(Participant 4, female 60–70, smartphone).*In addition, during ad-hock discussions with participants when performing technical support on the activity tracker, some participants stated that they compared activity tracker output with each other and found that interesting.

#### Activity tracker annoyances

This theme summarizes issues that participants found annoying about the activity tracker. Being annoyed with the activity tracker may reduce motivation to wear it. Sources of annoyance should therefore be identified and addressed if possible.

Technical challenges were a major source of annoyance, where participants experienced disconnects between their smartphone and activity tracker, and often found that the activity tracker was difficult to use without assistance. This was repeatedly mentioned during the interviews, prompting responses such as, “*negative about the watch … we got no instructions on how to use it*” *(Participant 1, male 60–70, smartphone),* “*a lot of information at once, considering I hadn’t used this [technology] before*” *(Participant 4, female 60–70, smartphone),* and *“I have struggled with the technical aspects” (Participant 13, male 60–70, smartphone).* Several participants mentioned that it could be helpful to have access to the instruction manual, to better understand both the complicated features and the more basic watch features. As stated by two participants: “*It was too complicated … but I didn’t spend too much time on it anyway … because we didn’t have the instruction manual” (Participant 10, female 70–80, smartphone),* and “*I miss instructions about the watch ... unsure how to set time*” *(Participant 7, male 70–80, smartphone).*

Activity tracker inaccuracies was also a major source of annoyance, and sleep feedback was repeatedly mentioned as a source of annoyance because of perceived inaccuracy. Two participants who had contradictory experiences may best describe this. One participant said, “*The only thing that annoys me … when I feel that I have slept very well … it reports how bad I have slept*” *(Participant 3, male 70–80, smartphone)*, and the other said, “*the sleep thing … I almost got annoyed sometimes … I woke several times per night, and sometimes I am out of bed three-four times ... and it reports that I have slept well*” *(Participant 12, female 70–80, no smartphone).*

One participant also noticed that the pulse sensors was not always accurate, and she got somewhat frustrated about this, stating that, “*I got very caught up in the [low] pulse measurements … resting at 39 [beats per minutes] during the day? I don’t get it*” *(Participant 4, female 60–70, smartphone).* Another participant also wondered about the accuracy during exercise sessions, and mention that, “*I wonder if the watch is correct … it’s not correct … much lower pulse … not even close*” *(Participant 5, male 70–80, no smartphone)*.

Interest in tracking activity was limited for some participants who did not own a smartphone (and we could *not* connect the activity tracker to their phone). When asked about whether they used the watch to track PA, one responded *“No, I didn’t, … we could have connected [the watch] to a smartphone, but I didn’t have [a smartphone*]” *(Participant 6, male 50–60, no smartphone).* Furthermore, when we asked if they missed any features on the activity tracker, only lack on direct feedback on PA metrics were mentioned by these participants. As stated by two participants without a smartphone: “*Sigh. Yes, steps*” *(Participant 16, female 70–80, no smartphone)* and “*Steps, … I am almost certain it is available on the watch*” *(Participant 14, male 60–70, no smartphone).* In addition, one participant, who owned a smartphone but where the connection between her smartphone and activity tracker was unstable and hampered data transfer, pointed out that this made it more complicated to use the activity tracker and said, “*It was hard to use the watch … I did not see the results as I wanted … I think those who saw their results on their phones got more out of it” (Participant 10, female 70–80, smartphone without successful connection)*.

#### Activity tracker improvements

The final theme captures suggestions that participants reported regarding the choice of activity tracker. Most participants were happy to wear the activity tracker during the intervention, both day and night, and reported no major issues with the day-to-day usage. However, some participants mentioned that the activity tracker could have been more attractive, and some felt it was too large and tight, prompting comments such as, “*It is [for instance] not good looking during the Christmas holiday*” *(Participant 4, female 60–70, smartphone*), “*I take it off when I dress up*” *(Participant 10, female 70–80, smartphone), “I have a more expensive watch I use when I want to look nice” (Participant 1, male 60–70, smartphone),* and “*It is a bit big … also tight*” *(Participant 1, male 60–70, smartphone).* Other participants were not too concerned about the design of the activity tracker, and one even made a point of saying “*I could not be bothered to wear another watch when at parties*” *(Participant 15, female 60–70, smartphone).*

Although the activity tracker had more features than we informed participants about, some pointed out that they knew other people with more advanced activity trackers with more interesting features. One participant said, “*My daughter has a more advanced [watch], with all possible features ... but it is of course more expensive*” *(Participant 13, male 60–70, smartphone).* On the other hand, another participant, who had a daughter with a less complex activity tracker, thought it would be better to use a less complicated activity tracker and commented that “*I liked it better* … *it was easier to use*” *(Participant 10, female 70–80, smartphone)*.

## Discussion

### Summary of findings

In this feasibility study with 12 months of PA recording, we analysed participant wear time, tested the Polar M430 validity in this sample, and reported participant experiences with long term usage. Wear time was high throughout the study. The Polar M430 over-reports steps (strong correlation) and MVPA (moderate correlation), and under reports TEE (moderate correlation). TEE had borderline acceptable error. Main *motivations* for increased wear time were that they were asked to do it and the ability to track activity progress. Regarding *usefulness*, most participants mainly used the activity tracker as a timepiece, but some also used it to measure heart rate and sleep tracking. In addition, reported positive effects were being more conscious about their day-to-day activity and improving their understanding of the effect of different activity types. Two major sources of *annoyance* were sleep- and -heart rate inaccuracy and limited instruction for use on the activity tracker. Suggestions for *improvement* were that the Polar M430 was big, unattractive, and too complicated to use.

### Participant characteristics

We invited 75 participants randomly selected from the seventh wave of the Tromsø Study. Since only 16 accepted the invitation, we included everyone who accepted, resulting in a sex skewed cohort of 70% men. All participants owned a mobile phone, but only 70% owned a smartphone. Smartphone penetration is lower in older age groups [44], which we also saw in this sample, as those owning a smartphone had a lower median age (64y vs 73y) compared to those who did own a smartphone.

### Polar M430

#### Wear time

In the present study, wear time was high, and most participants wore the activity tracker for the duration of the study. This high wear time is in alignment with a similar study, where Duignan et al. [45] conducted a shorter intervention study (3 months) in a younger sample (mean age: 23.4, SD: 2.8). In this study, 73% of participants still wore an activity tracker after 87 days, with an average wear time of 79 days (90%) among remaining participants. Reasons for loss of participants were mostly technical (e.g. data synchronizing) and loss of activity tracker. However, in a observational study by Hermsen et al. [46] they saw a slow exponential decline in wear time of a hip-worn Fitbit Zip, also mostly due to technical reasons, where only 16% still wore the activity tracker after 320 days. Although anecdotal, this indicates that being part of an intervention with close follow-up of participants increases wear time, as compared to studies where participants are only observed.

#### Polar M430 validity

In a systematic review of Polar activity trackers [47], we have previously reported that Polar activity trackers show mixed results depending on activity tracker, study setting, and study sample. Furthermore, compared to findings in a previous Polar M430 validation study [29], with a wider range of weight, height, and age, correlations were lower and MAPEs were higher in the present cohort.

The difference in results between the two validation studies shows that it is good practice to perform a separate validation study on participants with similar characteristics as the sample under study, when planning to use a consumer-based activity tracker in clinical research, as suggested by Phillips et al. [23]. The ActiGraph and the Polar M430 are worn of different locations, which may contribute to the large difference in MVPA and steps between devices. Certain activity types, e.g. stationary biking where hands are placed firmly on the bike’s handle, will result in more activity on the hip compared to the wrist. TEE is less affected by this difference as resting energy expenditure (energy consumed to maintain body functions at rest) is the main component of TEE and constitutes between 60 and 75% of TEE [48]. In addition, about 10% of TEE is expended from food digestion (dietary induced thermogenesis).

The Polar M430 is not a suitable replacement for the ActiGraph but can be used as a source of additional information for long term monitoring, for some variables.

### Participant perspective

#### Challenges and solutions

In the following, we discuss challenges and potential solutions, drawn from participants’ feedback together with experiences from the researcher perspective, and results from the objective data analyses.

##### Motivation and activity tracker usefulness

Most participants were enthusiastic about being invited to participate in the study. This was expressed repeatedly throughout the intervention during ad-hoc encounters. Because of this and because collecting data from the activity tracker was presented as an important part of the intervention, we do not find it surprising that wear time was high during the intervention. This is also in accordance with Duignan et al. [45] who achieved high wear time in a 3-month PA intervention. Wear time during follow-up was higher than expected, as the observational study by Hermsen et al. [46] showed high activity tracker attrition. However, the same study also showed that this attrition was lower in higher age groups, which may be part of the explanation of the high wear time in the present study.

Most users, when buying a new activity tracker, tend to stop using it after a few months, mostly due to loss of motivation [14, 49]. In the present study, only two of 16 stopped using the activity tracker after 6 months (i.e. intervention end). A major reason that participants in the present study wore the activity trackers for a full year, was because they were asked to wear it and they wanted to contribute to the study. This suggests external motivation and, at least for this group, may partly explain why activity tracker usage is not higher in the general population. About 20% of Americans use an activity tracker, with about 10% usage among people aged 55 and above [50]. While some reported annoyance with sleep and heart rate inaccuracies, we believe most participants were not too concerned with activity tracker accuracy, but more concerned about understanding how to use the activity tracker and having access to all collected data.

Similarly, we observed (during ad-hoc interactions with participants during the intervention) that some participants sometimes compared activity tracker output with each other. This may also indicate that having access to activity output for self-monitoring and being able to compare and compete with others was a possible source of motivation for prolonged wear time. This observation supports earlier findings which shows that activity tracker feedback can motivate PA participation in and of itself [14–16]. This effect must thus be considered when planning and analysing results of a PA intervention, to avoid ascribing increased PA participation to the intervention when the activity tracker itself may have been a major source of motivation. In addition, for participants who found the activity trackers useful during exercise sessions, and those who found it useful for learning which types of activity that were effective, it is likely that these features contributed to the increased wear time. It is apparent that activity tracker output is important for many, and unless there are specific reasons to *not* displaying these outputs, researchers should use an activity tracker that can show output that participants would find relevant to track their own progress (e.g. steps and/or minutes of MVPA).

##### Activity tracker annoyances

We found several sources of annoyance or nuisance among participants, where problems were mostly related to technical problems, activity tracker inaccuracy, and activity tracker complexity.

Technical problems during smartphone and activity tracker setup are likely to occur because of the large variation in participant phone models. It is therefore necessary to schedule enough time available for setup and have technically skilled personnel available who can resolve any issues directly. Too many technical problems may reduce participants motivation to wear an activity tracker. This is also suggested by Hermsen et al. [46] who found that the main reason (57%) for tracker attrition was related to technical problems. In addition, some participants did not bring their smartphone for the setup meeting, and several participants’ phones were out of power. Participants should have been reminded to bring a fully charged smartphone, and we should have brought charging equipment to the initial meeting. In addition, some participants lost their charging cable, and one misplaced the activity tracker for a period, showing that replacement equipment should also be available.

We did not specifically ask participants to clean the activity tracker regularly. Because of the long recording period, this caused the optical pulse sensor to become unclean and therefore unreliable. This sensor emits light onto the skin and estimates pulse by analysing changes in light waveform from the reflecting light. The reflecting light is affected by change in blood volume under the skin [51]. Annoyances about heart rate inaccuracy could have been avoided, at least partly, by instructing participants to clean the activity tracker regularly. In addition, the Polar M430 regularly misclassified sleep and non-wear time. Our main reason for selecting the Polar M430 was that it had a very good optical pulse sensor (according to Polar). However, we did not consider that being unable to disable sleep notifications could cause annoyance. We did not perform sleep validation on the Polar M430, which we (in retrospect) should have done to be able to inform participants about the possible inaccuracy of this metric.

Inaccuracy was mentioned as an individual issue and as a source of curiosity when participants compared activity tracker output between themselves and saw different results for the same activity. People are different and activity tracker output will differ between individuals. An additional possible source of variation may be activity tracker firmware, which is routinely updated by vendors. How updates affect activity tracker output are mostly company secrets. We therefore avoided updating the firmware unless we could update all activity trackers simultaneously. However, participants who connected the activity tracker to their smartphone were able to do this update more frequently, which resulted in several weeks where participants had different firmware. Activity tracker inaccuracy has also been identified by e.g. Hardcastle et al. [52] as a source of disappointment and false sense of achievement.

Several participants requested an easier way to view activity tracker output. The Polar M430 does not show daily step count automatically. This was annoying to several participants. This is also supported by Hardcastle et al. [52] who identified steps as the most popular feature of an activity tracker. Activity output would likely have been more accessible for participants if we had provided them with the instruction manual, which shows how to access this information. The main reasons for not providing the instruction manual were to prevent participants to change settings (e.g. turn on GPS tracking) or be affected by activity tracker output. However, since wearing an activity tracker is likely to only affect short term behaviour [14] we suggest providing participants with the instruction manual for long-term measurements. The importance of having access to the instruction manual and that lack of instructions are a source of annoyance, is also supported by previous studies on activity tracker use in older adults [53]. In the present study, some participants said that the Polar M430 was too complicated. However, in a study by McMahon et al. [54] on older adults using an activity tracker to increase PA, they showed that although older adults require more time to adopt new technology and needs more technical support [55], they found activity trackers easy to use and useful for PA self-tracking. Although this study used a Fitbit One, a less complex activity tracker compared to the Polar M430, adequate training in the present study would likely empower participants to use the activity tracker as intended.

##### Activity tracker improvements

Participant feedback regarding the activity tracker was mostly related to activity tracker design and available outputs. Hardcastle et al. [52] also found appearance to be important, and the Polar A300 (an earlier Polar model with similar design as the Polar M430) was found to be especially “bulky and clunky”. Similarly, Puri et al. [56] have also shown that aesthetics and comfort are important to increase activity tracker usage. When considering activity trackers in future studies, researchers should therefore consider appearance and usability, and not only price, accuracy, battery life, etc. Many vendors offer multiple versions of the same activity tracker, with different colours, shapes, and materials. Allowing participants to choose between multiple designs may increase wear time. This may be more important in a study with a younger population, as younger participants are more likely to own an activity tracker and may be resistant to replace it or start wearing an additional device. Similarly, because some participants said the activity tracker was too complicated and others said it was too simple, it could be beneficial to have more than one activity tracker available for participants to choose between, at least if the goal is to increase wear time. The drawback is that it is more complicated to compare activity levels between participants using different activity trackers.

#### Recommendations

From the above discussion, we have extracted the following recommendations that should be considered when planning and performing a study where participants are equipped with an activity tracker over a prolonged period. We have grouped recommendations into three phases: 1) the preparation and planning phase, 2) the setup and training phase, and 3) the recording phase.

##### Preparation and planning phase

- Budget for a technician who can provide technical support throughout the study and during follow-up.
- Offer activity trackers that can easily display relevant metrics, unless there are specific reasons not to display output.
- Allow participants to choose from multiple activity tracker designs, both in terms of complexity and appearance.
- Validate recent activity trackers in the relevant cohort if no such study exists, to identify acceptable activity trackers.
- Validate all metrics on the selected activity tracker and consider informing participants about untrustworthy metrics.

##### Activity tracker setup and participant training phase

- Provide adequate time for training and follow-up of participants.
- Remind participants to bring a fully charged smartphone (and bring charging equipment for common phones types) before connecting participants’ phones to their activity tracker.
- Instruct participants to clean the activity tracker regularly, to avoid inaccuracy in pulse measurements.
- Provide activity tracker instruction manual to participants, unless there are specific reasons not to.

##### Recording phase

- Keep close follow-up of participants to increase wear time.
- Have replacement activity trackers and charging equipment available.
- During study or follow-up; update activity tracker firmware simultaneously if possible.

#### Contribution to the literature

The most important contribution to the literature from this study is the identification of several important success factors that may increase wear time of an activity tracker, when provided to participants in a clinical study for PA tracking over a prolonged period. These factors have been summarized into a list of recommendations for clinical studies where similar methods of PA tracking are used. Following these recommendations may be timesaving for researchers, as well as reduce potential activity tracker annoyance among participants.

#### Strengths and limitations

The main aim of this paper was to identify factors that contributed to the wear time of the activity tracker. Study participants were recruited from a large ongoing population study, with a well-defined sample in terms of age, lifestyle habits, and health risks. This strength adds to the study’s transferability to similar population groups in similar societies [57]. Another strength is the use of a mixed methods approach and the long recording period, which allowed us to identify challenges from multiple perspectives and identify challenges that would not necessarily be detected in a study of shorter duration.

The main limitation is the limited transferability to other populations and age groups. Since participants were part of an intervention, desirability bias may have affected activity tracker wear time. This limits transferability of findings to other study designs. In addition, because only 16 participants were included, the variation in quantitative findings may be due to undetected differences in background characteristics. Participation, although low (21%), is as expected because intervention studies are unavoidably hampered by selection bias because participation demands high motivation and compliance. This challenge is further reinforced in studies that also require considerable efforts from participants, i.e. lifestyle interventions. In addition, older people often decline participation in PA interventions [58]. Acceptance assessment for the underlying feasibility study is addressed in Deraas et al. [28]. Further, participants were recruited from a population-based health study, and although the attendance was 72% in this age-group [59], this may introduce selection bias.

## Conclusions

In this study, long term activity tracker wear time was high. Results indicate that it is feasible to use a consumer-based activity tracker to measure PA over a longer period. Potential success factors for increased wear time includes providing adequate instructions on how to use the activity tracker, allowing participant to choose between different activity tracker designs (appearance and complexity), and offer activity trackers with accurate measurements. Validation studies on recent activity trackers may be needed for the target cohort, to identify such trackers.

## Data Availability

The data/transcripts used during the current study are available from the corresponding author on reasonable request.

## Abbreviations

AT: Activity tracker
EE: Energy expenditure
GPS: Global positioning system
MAPE: Mean absolute percentage error
MVPA: Moderate-to-vigorous physical activity
PA: Physical activity
RCT: Randomized controlled trial
TEE: Total energy expenditure
